# Advancing the quality of maternal, newborn, and child healthcare: insights from pilot hospitals in the Kyrgyz Republic

**DOI:** 10.7189/jogh.15.04256

**Published:** 2025-09-26

**Authors:** Nurshaim Tilenbaeva, Sagynbu Abduvalieva, Arsen Askerov, Dmitrii Beglitse, Masara Gapaeva, Anastasia Kisova, Oleg Kuzmenko, Khatuna Lomauri, Asel Orozalieva, Zaure Ospanova, Venera Shukurova, Olga Teplyakova, Dmitry Yasakov, Sophie Jullien, Martin Willi Weber, The Kyrgyz Republic Quality of Maternal, Newborn and Child Care Study Group, Dmitrii Beglitse, Khatuna Lomauri, Zaure Ospanova, Irina Stepanova, Olga Teplyakova, Anastasia Kisova, Masara Gapaeva, Dmitry Yasakov, Arsen Askerov, Sagynbu Abduvalieva, Venera Shukurova, Asel Orozalieva, Aziat Asanova

**Affiliations:** 1 World Health Organization Country Office in the Kyrgyz Republic, Bishkek, Kyrgyz Republic; 2 World Health Organization Athens Office on Quality of Care and Patient Safety, Athens, Greece; 3 Department of Neonatology, National Maternal, Newborn and Child Centre, Bishkek, Kyrgyz Republic; 4 Association of Obstetricians and Gynaecologists of the Kyrgyz Republic, Bishkek, Kyrgyz Republic; 5 Municipal maternal hospital No.1, Krasnodar, Russian Federation; 6 Gynaecology and Perinatology, National Medical Research Centre for Obstetrics, Moscow, Russian Federation; 7 Country Health Policies and Systems, World Health Organization Regional Office for Europe, Copenhagen, Denmark; 8 Department of Neonatology, Tbilisi State Medical University, Tbilisi, Georgia; 9 Kyrgyz Alliance of Midwives, Bishkek, Kyrgyz Republic; 10 Municipal polyclinic No.7, Astana, Kazakhstan; 11 Paediatric Department, Kyrgyz State Medical Institute of Continuous Education named after S.V. Daniyarov, Bishkek, Kyrgyz Republic; 12 National Medical Research Centre for Children’s Health, Moscow, Russian Federation

## Abstract

**Background:**

Maternal, newborn, and child mortality rates in the Kyrgyz Republic are high compared to other countries in the European Region of the World Health Organization (WHO). Global evidence suggests that at least half of the maternal and newborn deaths could be prevented with improved quality of healthcare. To address this, we undertook a quality improvement project over two years in ten pilot hospitals of the Kyrgyz Republic.

**Methods:**

We assessed the quality of care for maternal, newborn, and child health using WHO tools at the beginning and end of the project. We evaluated the availability and appropriate use of resources, case management, and key hospital policies. We used a standardised scoring system from 0 to 3, with colour coding scores and a display of trends (improved, deteriorated, remained the same). After the baseline assessment, we conducted a complex improvement process including the development of hospital quality improvement plans, updating clinical guidelines, training activities in priority topics, supportive supervision, and semi-annual collaborative quality improvement meetings between hospitals.

**Results:**

The baseline assessment revealed many areas of suboptimal care across the hospitals and technical areas. The endline assessment showed improvements in case management practices (baseline mean (x̄) = 1.6 *vs.* endline x̄ = 1.9) and policies and organisation of services (baseline x̄ = 1.7 *vs.* endline x̄ = 1.9). No improvement was achieved in hospital support services (baseline x̄ = 1.7 *vs.* endline x̄ = 1.8). Eight out of ten hospitals demonstrated overall improvement progress across categories; the two remaining hospitals showed no improvement.

**Conclusions:**

A complex intervention process focussed on updating clinical guidelines, selected capacity-building activities, supportive supervision, and semi-annual collaborative meetings led to quality improvements in maternal, newborn, and child health. The improvements achieved were still not reaching international standards, highlighting the need for a comprehensive and system-wide approach to quality improvement.

Over the past decades, the Kyrgyz Republic has achieved a significant reduction in maternal, newborn, and child mortality. Between 2001 and 2021, neonatal mortality decreased by 40% (from 19.9 to 11.9 per 1000), under-five mortality by 63% (from 47.4 to 17.4 per 1000), and maternal mortality by 42.5% (from 87 to 50 per 100 000) [1]. Despite this progress, mortality rates are still high compared to the average in the World Health Organization (WHO) European Region.

Globally, poor quality of care often leads to poor health outcomes. It is estimated that 50% of maternal deaths and 58% of newborn deaths could be prevented with improved quality of healthcare [2]. Quality of care is a central topic for Sustainable Development Goal target 3.8 on achieving universal health coverage, requiring access to health services that are safe, effective, and acceptable to all people. A review of the Kyrgyz Republic’s healthcare quality of care found that while many policies and mechanisms for quality improvement exist, they are often not fully implemented or integrated [3]. Quality is mainly driven by top-down command and external inspections rather than being embedded in clinical practice and training. External assessments focus more on control than on supportive improvement. Issues in healthcare delivery are not systematically addressed, and adverse outcomes are underreported. The unpublished observation of the Kyrgyz Republic’s healthcare system highlighted several critical issues, including insufficient funding, inefficient use of resources, and a lack of proper transportation systems for newborns, women in labour, and sick women. Hospitals and primary healthcare facilities are inadequately prepared for emergency care, with healthcare workers lacking the necessary skills and equipment. There is also a lack of effective monitoring tools for evaluating medical services, and an insufficient number of qualified medical personnel, especially in rural areas.

To address some of these challenges, we undertook a quality improvement project in ten hospitals of the Kyrgyz Republic from 2021 to 2023 through the project on ‘Improving the quality of hospital care to reduce maternal, newborn and child deaths and accelerate achievement of the Sustainable Development Goal health targets’. The project aimed to strengthen the national health system’s capacity to accelerate the reduction of preventable maternal, newborn, and child mortality by improving the quality of care.

Improving the quality of care often requires multifaceted strategies. Literature and past projects highlight capacity building among providers as a practical focus [4], which was recognised as the most capable of improvement during the project's duration. Alongside provider-focussed efforts, national-level actions – such as updating clinical guidelines and high-level advocacy measures to promote evidence-informed practices – supported broader systemic change. This contrasts with approaches that rely more heavily on infrastructure development. Alongside the Kyrgyz Republic, Tajikistan, Mongolia, and Vietnam also participated in the project. We aim to present the impact of the project on the quality of maternal, newborn, and child healthcare in the Kyrgyz Republic. Cross-country comparisons between the two central Asian project countries in the areas of maternal, newborn, and child health are presented elsewhere, as are the findings from the parallel project in Tajikistan [5-9].

## METHODS

### Target hospitals

The Ministry of Health of the Kyrgyz Republic identified ten pilot hospitals for the quality improvement project, out of 80 providing maternal, newborn, and child care in the country. Hospital selection focussed on geographic diversity, care level, delivery volume, absence of recent projects, motivated staff, and mentoring capacity. Project hospitals were primary, secondary, and tertiary level hospitals with capacities of 24–75 maternity beds, 1038–6778 deliveries per year for maternity hospitals, and 16–90 beds for paediatric hospitals.

### Data collection and assessment process

For the baseline (October 2021) and endline (October 2023) assessments, we used WHO assessment tools on hospital care for children [10] and hospital care for mothers and newborn babies [11]. We conducted the data sampling within hospitals in alignment with the given tools. The tools evaluated three main pillars: hospital support services, case management, and policies and organisation of services. We collected the data using direct observation, analysis of medical documentation, policies and procedures, clinical guidelines, standard operating procedures, inventory and operational reports, and interviews with staff, management, patients and care providers. The focus of the tools remained on the health system, and not on the individual, with a non-blaming, supportive approach.

### Data entry and calculation of trends of change

We conducted the endline assessment without the teams' knowledge of the baseline assessment scores. We transcribed the data into an Excel sheet and calculated the differences in the scores between baseline and endline assessment. We considered a change of >0.2 between the baseline and endline assessment score as improvement, while a≤−0.2 change was considered a deterioration of services. We considered all other values as ‘no change’. For the cut-offs, we followed the approach suggested in the WHO Quality Improvement manual to reflect the balance between possible random variation and documenting real improvement (or deterioration). [12]. We implemented several strategies to ensure data quality checks. During daily assessment team meetings, team leads, along with team members, cross-checked data for completeness, consistency, and accuracy. They also discussed any data entry errors to ensure the integrity of the collected information. After completing both baseline and endline assessments, they conducted two-day assessment synthesis workshops. These workshops aimed to verify, analyse, and interpret the data. They brought all teams together to ensure a comprehensive review and understanding of the data collected (Figure S1 in the **Online Supplementary Document**).

### Assessment teams and training of assessors

Multidisciplinary teams of national and international experts conducted both assessments. A team comprising at least two obstetricians, two midwives, two neonatologists, two paediatricians, and one paediatric nurse assessed each hospital. Team leads were international WHO consultants from other countries. Assessors underwent rigorous training, including a four-day programme on Effective Perinatal Care and the Pocket Book of Hospital Care for Children, followed by a three-day training on WHO assessment tools before the baseline assessment [13,14]. Additionally, the same group of assessors underwent a two-day refresher training on WHO assessment tools before the endline assessment. Exercises during the training helped to standardise the scoring.

### Scoring system

We used a standardised scoring system with scores ranging from 0 to 3. We used a ‘traffic light’ colour coding system to present the results in the form of heat charts. We used green for care that met international standards (*i.e.* little to no change needed for quality improvement), and yellow for care that needed some improvement to reach the standards (*i.e.* care is suboptimal but without a significant health hazard). Furthermore, we used orange for care that needed substantial improvement to reach the standards (*i.e.* suboptimal care with considerable health hazards), and red for care that needed substantial improvement (*i.e.* inadequate care and harmful practices with severe health hazards). The assessment teams summarised the mean (x̄) values for each assessment pillar and intervention hospital. Interviews with health workers and patients complemented numerical scores by capturing perceptions of quality of care. We provided the assessors with clear instructions on the scoring process, including the need for score justifications. We held daily meetings during each assessment to discuss, justify, and reach consensus on the scores. We assigned a score of >2.5 green, 1.8–2.4 yellow, 1–1.7 orange, and <1 red colour.

### Interventions

Following the baseline assessment in October 2021, we conducted a complex intervention process over two years, which included updating clinical guidelines, selecting capacity-building activities in technical areas and quality improvement methods, providing supportive supervision, and holding semi-annual collaborative meetings between participating hospitals (Figure S2 in the **Online Supplementary Document**). The project interventions were localised in pilot health facilities through capacity-building activities, primarily focussed on on-the-job-training in Effective Perinatal Care, the Pocket Book of Hospital Care for Children. We conducted regular supportive supervision visits to facilitate the practical implementation of knowledge and skills. All pilot hospitals received the same set of training packages, although over different time spans. Additionally, we provided the nurses with on-the-job training on newly developed standard operating procedures to align with the Pocket Book on Hospital Care for Children. Some interventions targeted national-level efforts, which were essential for achieving sustainable changes from a health system perspective. These activities included the development and updating of several clinical guidelines in maternal, newborn, and child health, high-level national policy dialogues on evidence-informed maternal, newborn, and child death surveillance and response approaches, and national roundtable discussions to address pressing issues related to the availability, use, and maintenance of medical equipment.

The intervention process aligned with the WHO Regional framework for improving the quality of care for reproductive, maternal, neonatal, child, and adolescent health. The National Steering Group was established to oversee the project implementation, and the Ministry of Health chaired it.

Pilot hospitals developed their quality improvement plans based on the results of the baseline assessment. The plans were continuously updated after each supportive supervision visit and semi-annual collaborative meeting. Hospital teams prioritised one to three priority issues to address in each biennium. Complementary activities of the project included pilot testing of a new modality of work for paediatric nurses, shifting from a traditional task-oriented to a patient-centred mode, situation analysis on availability, use, and maintenance of medical equipment in pilot hospitals, and advocacy work to address a punitive environment of the quality culture in the setting.

## RESULTS

### Policies and organisation of services

**Figure 1:**
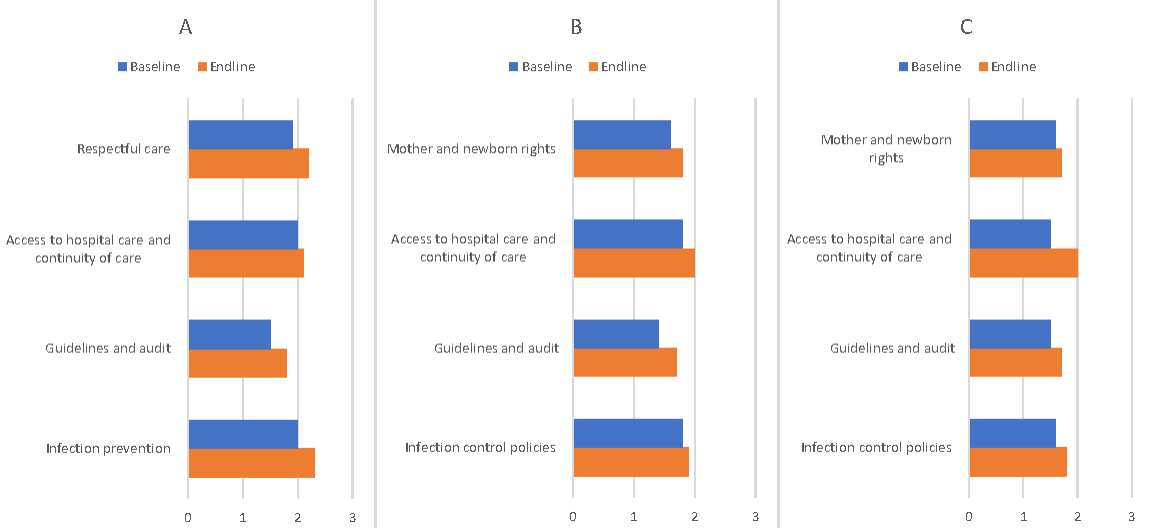
Policies and organisation of services. **Panel A**. Paediatric care. **Panel B**. Maternal care. **Panel C**. Newborn care.

Policies and organisation of services showed a tendency for improvement across areas (**Figure 1**, Panels A–C; Figure S1, Panel C in the **Online Supplementary Document**). Policies and organisation of services for paediatric care were considerably more advanced at the baseline (x̄ = 1.8) compared to maternal and newborn care (x̄ = 1.6). Guidelines and audit were improved across areas (baseline x̄ = 1.5 *vs.* endline x̄ = 1.8). Infection prevention and control mainly were improved for paediatric care (baseline x̄ = 1.8 *vs.* endline x̄ = 2.1) (**Figure 1**, Panel A). Five out of nine paediatric hospitals were able to meet international standards on infection control by the endline. Improvements were achieved in the use of gloves, laundry arrangements, waste management, and medical sterilisation.

Access to hospital care and continuity of care were improved for maternal and newborn care (**Figure 1**, Panels B and C). Respectful care was only enhanced for paediatric care (baseline x̄ = 1.9 *vs.* endline x̄ = 2.2), and remained unchanged for maternal (baseline x̄ = 1.6 *vs.* endline x̄ = 1.8) and newborn care (baseline x̄ = 1.6 *vs.* endline x̄ = 1.7). Three out of nine paediatric hospitals met international standards on respectful care. The endline assessment showed a reduction of unreasonably painful and invasive procedures; parents were allowed to stay with their children, and mothers could breastfeed their children on demand.

Overall, three out of ten hospitals showed considerable improvement in policies and organisation of services across the areas. One hospital demonstrated deterioration, and the other hospitals had different improvement levels across technical areas.

### Case management

Case management practices improved at different levels across areas. Clinical practices in paediatric (x̄ difference = 0.3) and newborn care (x̄ difference = 0.4) improved more than in maternal care (x̄ difference = 0.2) (Figure S1, Panel B in the **Online Supplementary Document**). The improvement was observed in most areas in paediatric (n = 6/N = 8) and neonatal care (n = 3/N = 4), but only in one area in maternal care (N = 3). However, the baseline of the quality of maternal care (x̄ = 1.8) had higher scores than paediatric (x̄ = 1.6) and newborn care (x̄ = 1.5) (Figure S1, Panel B in the **Online Supplementary Document**).

### Paediatric care

**Figure 2:**
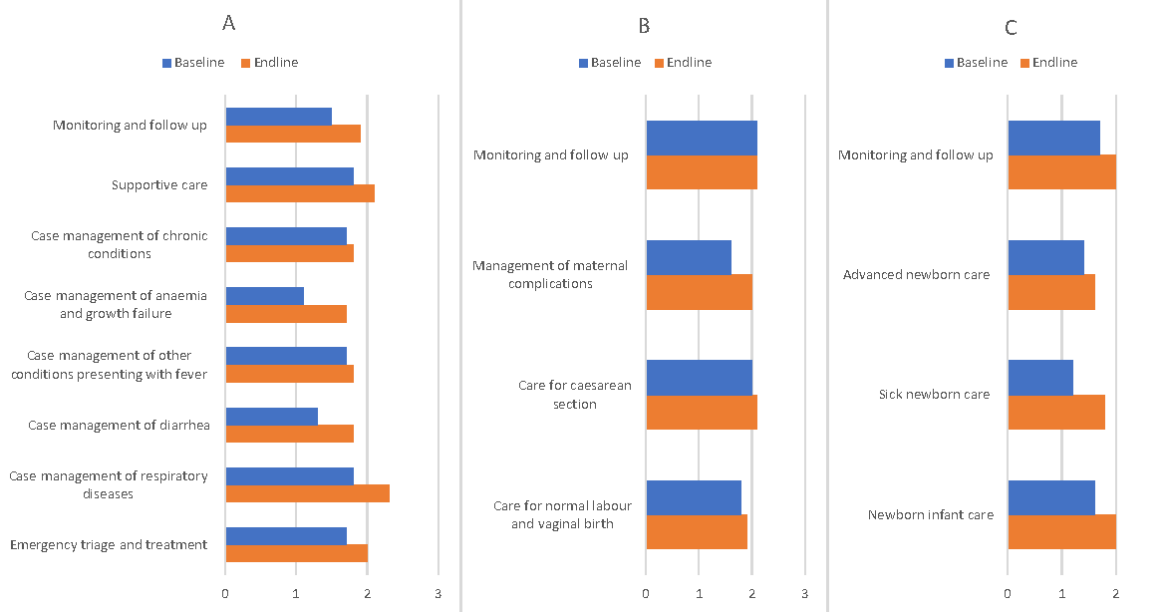
Case management. **Panel A**. Paediatric care. **Panel B**. Maternal care. **Panel C**. Newborn care.

Case management practices for paediatric care improved across all hospitals. Eight out of nine hospitals demonstrated progress in care practices (Figure S1, Panel B in the **Online Supplementary Document**). Despite improvements, the management of anaemia and growth failure was still the weakest area across the hospitals (baseline x̄ = 1.1 *vs.* endline x̄ = 1.7). Investigations for suspected pneumonia (x̄ = 2.7) and management of children with bronchial obstruction (x̄ = 2.6) were identified as practices aligned with international standards by the endline assessment (Figure S3 in the **Online Supplementary Document**). Assessment and screening for pneumonia, use of clinical classification of pneumonia, appropriate selection of antibiotics, and use of oxygen therapy were improved in most hospitals. Case management of respiratory infections, diarrhoea, anaemia, and growth failure improved across hospitals (**Figure 2**, Panel A).

Case management of chronic conditions and conditions presenting with fever remained unchanged (baseline x̄ = 1.7 *vs.* endline x̄ = 1.8). Unjustified hospitalisation remained an issue, increasing from 40% to 56%, with a large proportion of visits to hospital admission rooms bypassing primary health facilities. The detailed evaluation of the results is described elsewhere [8].

### Maternal care

Case management of maternal clinical practices did not improve in general (baseline x̄ = 1.8 *vs.* endline x̄ = 2.0), except for management of maternal complications (baseline x̄ = 1.6 *vs.* endline x̄ = 2.0) (**Figure 2**, Panel B; Figure S4, Panel A in the **Online Supplementary Document**). This category had both areas which reached international standards (*i.e.* appropriate medicine use) (endline x̄ = 2.8) and an area with the most inadequate care (*i.e.* intrauterine growth restriction) (endline x̄ = −0.8). Management of postpartum haemorrhage, prolonged labour, preeclampsia, and eclampsia were improved across the hospitals. Improved referral, use of magnesium sulphate, and vaginal birth as a preferred choice contributed to the given improvements. Overdiagnosis of preeclampsia and severe preeclampsia remained a challenge, leading to unnecessary hospitalisation, overmedicalisation, and inappropriate timing of delivery.

Despite the absence of overall improvement in care for caesarean section, the surgical technique for caesarean section achieved international standards by the endline assessment (endline x̄ = 2.6) (Figure S4, Panel B in the **Online Supplementary Document**). Further improvements were demonstrated for care at admission for normal labour and vaginal birth, prevention of infections, and early puerperium management.

Six out of nine maternity hospitals demonstrated improvement in care practices across the areas, while three hospitals did not improve the quality of maternal care (Figure S1, Panel B in the **Online Supplementary Document**). The detailed evaluation and discussion of the results are described elsewhere [6].

### Newborn care

Case management of newborns achieved considerable progress across the assessed clinical practices, especially in routine newborn care, sick newborn care, and monitoring and follow-up (**Figure 2**, Panel C). Care at birth and during the first two hours of life improved from baseline (x̄ = 1.7) to endline (x̄ = 2.1) (Figure S5, Panel A in the **Online Supplementary Document**). Challenges with inconsistent monitoring, substandard documentation of critical parameters, and a lack of linkage between nurse records and overall medical records persisted.

Advanced newborn care remained unchanged (baseline x̄ = 1.4 *vs.* endline x̄ = 1.6). Suboptimal practices were observed in several areas, including enteral nutrition, parenteral infusions, treatment of respiratory problems, pain avoidance and control, neonatal developmental care, and transport of critical infants. Improvements were achieved in clinical records for the newborn intensive care unit (baseline x̄ = 1.4 *vs.* endline x̄ = 1.7), nutritional outcome indicators (baseline x̄ = 0.8 *vs.* endline x̄ = 1.4), other specific conditions (baseline x̄ = 0.9 *vs.* endline x̄ = 1.4), appropriate use of medicines (baseline x̄ = 0.9 *vs.* endline x̄ = 1.9), communication with parents (baseline x̄ = 2.1 *vs.* endline x̄ = 2.6), and quality improvement and audit (baseline x̄ = 1.4 *vs.* endline x̄ = 1.7) (Figure S5, Panel B in the **Online Supplementary Document**).

Overall, six out of nine hospitals demonstrated considerable improvements across newborn care practices. Two out of nine hospitals did not achieve any progress, whereas one hospital showed lower scores at the endline (Figure S1, Panel B in the **Online Supplementary Document**). The detailed evaluation and discussion of the results are described elsewhere [7].

### Hospital support services

**Figure 3:**
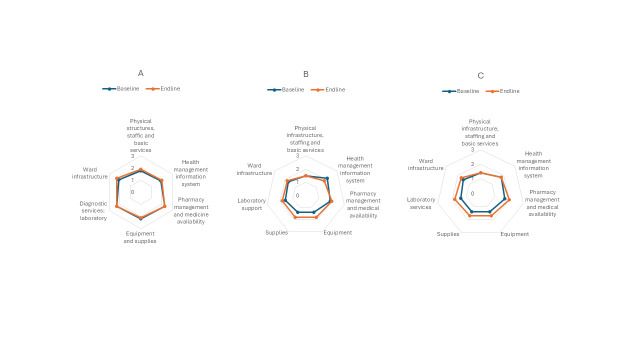
Hospital support services. **Panel A**. Paediatric care. **Panel B**. Maternal care. **Panel C**. Newborn care.

Overall, hospital support services showed no significant improvements and remained suboptimal for newborn care, requiring further enhancements to meet standards of care for paediatric and maternal care (**Figure 3**, Panels A–C; Figure S1, Panel A, Figure S6, Panels A and B in the **Online Supplementary Document**). Support services for paediatric care were the highest at the baseline (x̄ = 2.1), with no improvements observed at the endline (x̄ = 2.1) (**Figure 3**, Panel A). Hospital support services for newborn care had lower scores than maternal and paediatric care but improved the most (baseline x̄ = 1.4 *vs.* endline x̄ = 1.6) (**Figure 3**, Panel C; Figure S6, Panels A and B in the **Online Supplementary Document**).

The physical infrastructure, staffing and basic services, along with the health management information system, had no changes from baseline (x̄ = 1.5) to endline (x̄ = 1.5) across all the areas. Almost all paediatric wards lacked cribs, resulting in mothers sharing beds with their babies. The same situation was observed for changing tables in the wards and privacy areas for parents and older children. Hospitals struggled with having enough essential equipment and consumables (*i.e.* electric suction devices, spacers, oxygen masks, catheters, nasogastric tubes of all sizes, child-sized air ducts, otoscopes, pulse oximeters, and medical device sensor sizes suitable for small children).

Two out of ten hospitals did not achieve considerable improvement across the different areas.

## DISCUSSION

### Main findings

We assessed the effectiveness of a complex set of quality improvement interventions implemented over two years to improve the quality of maternal, newborn, and paediatric care in ten hospitals of the Kyrgyz Republic. Improvements were made in case management, policies and organisation of services across the hospitals, and technical areas, with no improvements observed in hospital support services. This can be explained by the focus of project initiatives on capacity building of care providers, with minimal to no inputs made to improve physical infrastructure, equipment, supplies, and the health management information system.

Additionally, there were variations in performance across different technical areas, with paediatric services overall showing more advanced outcomes in the assessment dimensions. This may be partly due to the long-term effects of a paediatric quality improvement project in Central Asia, which included the Kyrgyz Republic. The Pocket Book on Hospital Care for Children was introduced in the country in 2013 as part of the project, serving as the foundation for training and standard setting. [15] Furthermore, future studies could be beneficial in examining the role of earlier interventions, general resource allocation differences within public funding, policy and advocacy, and family and community engagement in quality improvement, to understand these differences.

A study applying the same assessment methodology and a set of interventions in the Republic of Tajikistan showed improvements across all domains [9]. In 2021, most pilot hospitals fell within the red and yellow categories, showing below-standard performance. The endline assessment in 2023 showed progress to the yellow or green categories, demonstrating improved quality of care. This could be due to lower scores at the baseline for hospital support services and, thus, greater scope for improvement in the parallel study.

### Infrastructure and human resources

Pilot hospitals largely lacked the required infrastructure, equipment, and supplies at both the baseline and endline assessments, which is expected since no infrastructural inputs were provided within the project’s framework. Research consistently highlights infrastructure as an enabler for the provision of quality of care [16]. However, solely investing in infrastructure will not improve the quality of care unless a sustainable system for its use and maintenance is firmly established. The unpublished observation on use, availability, and management of medical equipment in pilot hospitals revealed that only 62% of the available basic equipment was functional, 50% of hospitals lacked medical technicians, and none of the hospitals allocated the minimally required 5% of annual hospital budget for equipment maintenance costs. The findings were presented at the National roundtable with key stakeholders to advocate for addressing systemic barriers related to the infrastructural capacities of healthcare facilities.

Human resources and staffing for quality of care require further attention. The Kyrgyz Republic has one of the lowest densities of medical doctors, nurses, and midwives per 100 000 population in the WHO European region [17]. These issues are not unique to the Kyrgyz Republic; many lower-middle-income countries encounter similar barriers within their health systems. Lack of material and human resources, as well as problems with communication and information sharing, are common [18]. Another study suggested that despite the lack of basic infrastructure and supplies necessary for quality of care in resource-limited settings, the structures of the collaboratives, with open sharing of ideas during learning sessions, encouraged health workers to use data effectively to advocate for solutions [19]. The culture of databased self-advocacy provided an alternative to strategies used to bridge the structural gaps.

### Collaborative approaches

Semi-annual quality improvement meetings were an integral part of the quality improvement initiative in the Kyrgyz Republic, with four held over the course of the project. These meetings promoted collaboration across hospitals and engaged hospital quality committee members along with maternal, newborn, and child care providers. Evidence from East Africa has shown that facility-based quality improvement collaboratives are an effective tool for improving quality of care [20], as they facilitate peer learning. In Kenya and Uganda, a collaborative approach, combined with clinical and teamwork skills training, data strengthening, and the WHO Safe Childbirth Checklist, resulted in improved neonatal outcomes. Health workers appreciated cross-facility learning, friendly competition, and integration of quality improvement practices into routine care.

### Impact of hospital management and leadership

Eight out of ten hospitals demonstrated overall improvement progress across categories, whereas two showed no improvement. There was no evident link between the size of hospitals, the level of service provision, the number of beds, or the number of deliveries and the progress in quality improvement over the two years. Hospitals with more stable and engaged hospital management showed greater improvement over hospitals with frequent management changes. The two hospitals that did not achieve considerable progress experienced frequent changes in hospital management, leading to inconsistent or absent leadership participation in collaborative quality improvement meetings. Hospitals with the greatest improvement results had highly supportive hospital management observed during the collaborative meetings. Leadership stability and capacity were highlighted as one of the key elements for meso-level quality improvements [5]. The literature suggests that managers’ time spent, engagement, and work can influence quality outcomes, processes, and overall performance [21]. Technical areas within the same hospitals were at different levels and paces for improvement, suggesting that middle managers play an essential role in bridging between hospital administration (*i.e.* strategies) and frontline staff (*i.e.* day-to-day care practices). The evaluation from the literature highlights several characteristics and skills of leaders that are specific to quality improvement programmes [22], such as enthusiasm about the quality improvement methodology, technical skills to run quality improvement programmes, views that quality improvement is a tool to support what healthcare providers are supposed to do, and fostering a learning culture among team members.

### Learning and quality culture

Learning culture is a prerequisite for quality and safety cultures. We found that guidelines and audit categories were among the weakest in the policies and organisation of services area. Improvement audits were largely not in line with the WHO-recommended approaches for quality improvement, despite institutionalisation and pilot testing of Confidential Enquiries into Maternal Deaths, Near-miss Case Reviews at the hospital level, and Perinatal Audits in the country [23,24]. Unpublished observation suggests that there is a prevailing practice of person-focussed failure approaches in the country, which could be one of the greatest challenges for building an enabling quality culture for improvements. A punitive culture is demoralising, counter-productive, and negatively impacting on the quality of care [25]. Supportive supervisions within the project were promoted mainly to address person-focussed failure approaches. They aimed to support healthcare providers in applying the knowledge gained from training programmes to real-life settings. Five rounds of supportive supervision were conducted throughout the project duration. Another study from the Kyrgyz Republic suggested that periodic supportive supervision for one year after a training course improved both adherence to WHO guidelines on hospital care for children and overall quality of paediatric care [26].

### System-wide approach for quality improvement

Despite considerable improvements in two years, many care practices were still not reaching international standards. Improvements in case management, policies, and organisation of services were often not backed up by hospital support services. A system-wide approach is necessary to enhance the quality of care. Addressing the quality deficits in countries requires expansion of the solution space from micro-level to meso- and macro-levels [2]. Quality improvement emerges from the interactions of various health system functions. These include well-trained and motivated healthcare workers, accessible and well-equipped facilities, and information systems that monitor and drive better care. Financing mechanisms and good governance play a crucial role in enabling and encouraging quality care. Effective interaction of these functions ensures the sustainability of achieved improvements.

### Long-term benefits and sustainability

Another study evaluating the long-term benefits of the quality of paediatric hospital care initiative 2012–15 in the Kyrgyz Republic showed that improvements were sustained after seven years in five out of six indicators [27]. The same interventions had less effect in the Republic of Tajikistan in the short term, and the improvement was not always sustained until 2021. They concluded that the favourable results in the Kyrgyz Republic can be attributed to better performance of the health system's functions.

Change takes time, and the quality improvement process will need to be consistently continued to achieve better progress. Small changes must be appreciated as they have the potential to make significant improvements. Support service deficits can indeed undermine the overall impact of the intervention. When healthcare providers lack the necessary support, such as adequate remuneration, well-equipped infrastructure, and access to essential diagnostic tests, medicines, devices, and technologies, their ability to deliver high-quality care is compromised. Additionally, a punitive culture within the healthcare system can further hinder progress. When healthcare workers fear punitive measures rather than receiving supportive supervision, it discourages open communication and continuous improvement despite capacity-building efforts. Therefore, to enhance the effectiveness and sustainability of future project iterations, those considerations should be prioritised. The project targeted these issues at the national level by leveraging and disseminating new and existing evidence through advocacy events at the highest level. However, further deliberate actions are required, including shifts in legal consideration related to punitive measures that jeopardise quality improvement. Ensuring that legal frameworks facilitate rather than obstruct quality improvement efforts is essential for achieving lasting change.

### Limitations and strengths

The absence of control hospitals is a limitation of the study; thus, the impact of project interventions might lead to overestimation towards improvements. We cannot be certain that the improvements were due to the interventions. However, this limitation was likely reduced through assessor training, maintaining consistent team composition, score cross-checking and validation, and international oversight. Additionally, the complementary implementation research conducted over the course of the project with a control arm addressed this limitation and reinforced findings of the given study [28]. In addition, the use of the standardised WHO quality of care assessment tools focussing on progress indicators performed by teams led by international assessors, deliberate capacity building of assessors on assessment tools, effective perinatal care, and the Pocket book of hospital care for children strengthened the study.

## CONCLUSIONS

The complex intervention process, which focussed on updating clinical guidelines, selected capacity-building activities in priority technical areas, quality improvement methods, supportive supervision, and semi-annual collaborative meetings, can lead to quality improvements in maternal, newborn, and child health. We believe that the approaches should be scaled up to other hospitals, with a potential expansion to primary healthcare settings. A health system’s lens needs to be applied to achieve sustainability and further quality improvement. A focus is required to build an enabling quality culture across all aspects of the health system, which encourages learning and improvement by addressing root causes of the challenges for quality of care. The continuous efforts must also reflect on the shortcomings and contextual barriers, such as limited infrastructure investment and persistent punitive norms, to provide a comprehensive understanding of the systemic issues at play.

## Additional material


Online Supplementary Document
