# Supplementary material for: Advancing the quality of maternal, newborn, and child healthcare: insights from pilot hospitals in the Kyrgyz Republic
Source: J Glob Health. 2025 Sep 26;15:04256. doi: 10.7189/jogh.15.04256 (PMC12467440; doi:10.7189/jogh.15.04256)

Supplement to: Tilenbaeva N, Abduvalieva S, Askerov A, Beglitse D, Gapaeva M, Jullien S, Kisova A, Kuzmenko O, Lomauri K, Orozalieva A, Ospanova Z, Shukurova V, Teplyakova O, Yasakov D, Weber MW; The Kyrgyz Republic Quality of Maternal, Newborn and Child Care Study Group. Advancing the quality of maternal, newborn, and child healthcare: insights from pilot hospitals in the Kyrgyz Republic. J Glob Health. 2025;15:04256.

Figure S1 in the **Online Supplementary Document**. Heat chart: baseline and endline assessment results[1]

Panel A. Hospital support services

HOSPITAL SUPPORT SERVICES

HOSPITALS

		1			2			3			4			5			6			7			8			9			10			Summary		
		Baseline	Endline	Difference	Baseline	Endline	Difference	Baseline	Endline	Difference	Baseline	Endline	Difference	Baseline	Endline	Difference	Baseline	Endline	Difference	Baseline	Endline	Difference	Baseline	Endline	Difference	Baseline	Endline	Difference	Baseline	Endline	Difference	Baseline	Endline	Difference
	**Pedaitric care**																																	
1.1	Physical structures, staffing and basic services	1.5	1.8	↑ 0.3	1.5	2.1	↑ 0.6	1.3	1.0	↓ -0.3	2.6	2.7	→ 0.1	2.0	2.1	→ 0.1	2.1	2.2	→ 0.1	1.3	1.4	→ 0.1	2.0	2.0	→ 0.0	1.8	1.8	→ 0.0				1.8	1.9	→ 0.1
1.2	Statistics, health management information systems and medical records	3.0	2.2	↓ -0.8	1.5	2.0	↑ 0.5	2.2	1.4	↓ -0.8	1.7	2.1	↑ 0.4	1.9	2.1	→ 0.2	1.9	2.0	→ 0.1	1.8	2.0	→ 0.2	2.0	2.2	→ 0.2	1.2	1.8	↑ 0.6				1.9	2	→ 0.1
1.3	Pharmacy management and medicine availability	2.5	1.9	↓ -0.6	2.5	2.0	↓ -0.5	2.0	2.0	→ 0.0	2.8	2.8	→ 0.0	3.0	2.7	↓ -0.3	2.8	2.6	→ -0.2	2.0	2.7	↑ 0.7	1.4	2.1	↑ 0.7	1.5	2.3	↑ 0.8				2.3	2.3	→ 0.0
1.4	Equipment and supplies	2.2	1.3	↓ -0.9	2.0	2.0	→ 0.0	2.3	1.0	↓ -1.3	2.5	2.7	↑ 0.2	2.3	2.5	→ 0.2	2.3	2.5	→ 0.2	2.0	2.0	→ 0.0	1.8	2.3	↑ 0.5	2.3	2.5	→ 0.2				2.2	2.1	→ -0.1
1.5	Diagnostic services: laboratory	2.0	1.8	→ -0.2	2.5	2.0	↓ -0.5	2.6	2.0	↓ -0.6	2.5	2.6	→ 0.1	3.0	2.6	↓ -0.4	3.0	2.8	→ -0.2	1.8	2.3	↑ 0.5	2.0	2.4	↑ 0.4	1.5	2.3	↑ 0.8				2.3	2.3	→ 0.0
1.6	Ward infrastructure	2.0	2.1	→ 0.1	1.5	2.1	↑ 0.6	2.0	1.5	↓ -0.5	2.4	2.5	→ 0.1	2.3	2.5	→ 0.2	2.6	2.8	→ 0.2	2.0	2.4	↑ 0.4	2.0	2.4	↑ 0.4	2.0	2.1	→ 0.1				2.1	2.3	→ 0.2
	**Maternal care**																																	
1.1	Physical infratructure, staffing, and basic services	1.0	1.3	↑ 0.3	1.3	1.7	↑ 0.4	1.0	1.0	→ 0.0				1.6	1.6	→ 0.0	2.3	1.6	↓ -0.7	1.3	1.6	↑ 0.3	1.7	1.0	↓ -0.7	1.7	1.3	↓ -0.4	2.0	2.3	↑ 0.3	1.5	1.5	→ 0.0
1.2	Statistics, health management information systems and medical records	1.6	1.2	↓ -0.4	1.8	1.8	→ 0.0	2.0	1.4	↓ -0.6				2.6	2.2	↓ -0.4	2.8	2.0	↓ -0.8	2.0	2.0	→ 0.0	1.8	1.6	→ -0.2	2.0	2.0	→ 0.0	2.0	2.4	↑ 0.4	2.1	1.8	↓ -0.3
1.3	Pharmacy management and medicine availability	1.6	1.9	↑ 0.3	1.6	2.3	↑ 0.7	1.0	1.6	↑ 0.6				2.4	2.4	→ 0.0	2.4	2.3	→ -0.1	2.0	2.0	→ 0.0	1.9	1.6	↓ -0.3	1.8	1.7	→ -0.1	2.3	2.2	→ -0.1	1.9	2	→ 0.1
1.4	Equipment	1.0	1.8	↑ 0.8	1.0	2.0	↑ 1.0	1.2	2.0	↑ 0.8				2.0	2.0	→ 0.0	1.2	2.0	↑ 0.8	1.8	1.5	↓ -0.3	1.0	1.5	↑ 0.5	1.2	1.3	→ 0.1	2.0	1.8	↓ -0.3	1.4	1.8	↑ 0.4
1.5	Supplies	1.0	1.8	↑ 0.8	1.0	2.0	↑ 1.0	1.2	2.0	↑ 0.8				2.0	2.0	→ 0.0	1.2	2.0	↑ 0.8	1.8	1.5	↓ -0.3	1.0	1.5	↑ 0.5	1.2	1.3	→ 0.1	2.0	1.8	↓ -0.3	1.4	1.8	↑ 0.4
1.6	Laboratory support	0.6	1.7	↑ 1.1	1.6	2.0	↑ 0.4	0.7	1.4	↑ 0.7				2.0	1.8	→ -0.2	2.1	2.1	→ 0.0	1.6	1.8	→ 0.2	2.0	2.0	→ 0.0	2.1	1.7	↓ -0.4	2.0	1.8	→ -0.2	1.6	1.8	→ 0.2
1.7	Ward infrastructure	1.6	1.6	→ 0	1.5	1.9	↑ 0.4	0.8	1.5	↑ 0.7				1.8	1.9	→ 0.1	2.6	2.0	↓ -0.6	2.1	2.0	→ -0.1	1.5	1.3	→ -0.2	2.0	1.6	↓ -0.4	1.8	2.1	↑ 0.4	1.7	1.8	→ 0.1
	**Neonatal care**																																	
1.1	Physical infratructure, staffing, and basic services	1.0	1.3	↑ 0.3	1.0	1.7	↑ 0.7	1.0	1.0	→ 0.0				1.6	1.6	→ 0.0	2.3	1.3	↓ -1.0	1.3	1.3	→ 0.0	1.7	1.0	↓ -0.7	1.0	1.3	↑ 0.3	2.0	2.3	↑ 0.3	1.3	1.3	→ 0.0
1.2	Statistics, health management information systems and medical records	1.0	1.2	→ 0.2	1.8	1.8	→ 0.0	1.8	1.4	↓ -0.4				2.6	2.2	↓ -0.4	2.8	2.0	↓ -0.8	1.4	2.0	↑ 0.6	1.6	1.4	→ -0.2	1.0	2.0	↑ 1.0	2.0	2.4	↑ 0.4	1.7	1.7	→ 0.0
1.3	Pharmacy management and medicine availability	1.0	1.9	↑ 0.9	1.0	2.3	↑ 1.3	1.0	1.6	↑ 0.6				2.2	2.4	→ 0.2	2.4	2.3	→ -0.1	1.8	2.0	→ 0.2	2.0	1.6	↓ -0.4	2.0	1.7	↓ -0.3	2.3	2.2	→ -0.1	1.63	1.79	→ 0.2
1.4	Equipment	1.0	1.8	↑ 0.8	1.0	2.0	↑ 1.0	0.7	1.5	↑ 0.8				2.0	2.0	→ 0.0	1.0	1.8	↑ 0.8	2.0	2.0	→ 0.0	1.0	1.5	↑ 0.5	1.0	1.3	↑ 0.3	3.0	1.8	↓ -1.3	1.30	1.53	↑ 0.2
1.5	Supplies	1.0	1.8	↑ 0.8	1.0	2.0	↑ 1.0	0.7	1.5	↑ 0.8				2.0	2.0	→ 0.0	1.0	1.8	↑ 0.8	2.0	2.0	→ 0.0	1.0	1.5	↑ 0.5	1.0	1.3	↑ 0.3	3.0	1.8	↓ -1.3	1.30	1.53	↑ 0.2
1.6	Laboratory support	0.6	1.7	↑ 1.1	0.6	2.0	↑ 1.4	0.7	1.4	↑ 0.7				2.0	1.8	→ -0.2	2.1	2.1	→ 0.0	1.5	1.8	↑ 0.3	2.0	2.0	→ 0.0	1.0	1.7	↑ 0.7	2.0	1.8	→ -0.2	1.32	1.62	↑ 0.3
1.7	Ward infrastructure	0.9	1.6	↑ 0.7	1.4	1.9	↑ 0.5	0.0	1.2	↑ 1.2				2.1	1.8	↓ -0.4	3.0	2.0	↓ -1.0	1.9	2.0	→ 0.1	1.6	1.0	↓ -0.6	1.0	1.8	↑ 0.8	1.9	2.1	↑ 0.23	1.4	1.5	→ 0.1

Panel B. Case management

CASE MANAGEMENT

HOSPITALS

		1			2			3			4			5			6			7			8			9			10			Summary		
		Baseline	Endline	Difference	Baseline	Endline	Difference	Baseline	Endline	Difference	Baseline	Endline	Difference	Baseline	Endline	Difference	Baseline	Endline	Difference	Baseline	Endline	Difference	Baseline	Endline	Difference	Baseline	Endline	Difference	Baseline	Endline	Difference	Baseline	Endline	Difference
	**Pediatric care**																																	
1	Emergency triage and treatment	1.4	1.8	↑ 0.4	2.5	1.7	↓ -0.8	1.2	1.0	↓ -0.21	1.5	2.2	↑ 0.7	1.7	2.0	↑ 0.3	1.9	2.0	→ 0.1	1.6	2.0	↑ 0.4	1.7	2.1	↑ 0.4	2.0	2.9	↑ 0.9				1.7	2.0	↑ 0.2
2	Case management of respiratory diseases	2.1	2.2	→ 0.1	2.5	2.7	→ 0.2	1.5	1.8	↑ 0.4	1.8	2.7	↑ 1.0	1.6	2.2	↑ 0.6	1.9	2.2	↑ 0.4	2.0	2.1	→ 0.1	1.5	2.3	↑ 0.8	1.8	2.0	→ 0.2				1.8	2.3	↑ 0.4
3	Case management of diarrhoea	1.0	1.7	↑ 0.7	1.0	1.5	↑ 0.5				1.4			1.3	1.5	→ 0.2	1.7	1.8	→ 0.1	1.5	2.0	↑ 0.5	1.4			1.0	2.1	↑ 1.1				1.3	1.8	↑ 0.5
4	Case management of other conditions presenting with fever	2.3	1.5	↓ -0.8	1.5	1.5	→ 0.0	1.4	1.8	↑ 0.4	1.8	1.7	→ -0.1	1.4	1.8	↑ 0.4	1.7	1.4	↓ -0.3	2.0	2.1	→ 0.1	2.0	2.3	↑ 0.3	1.1	1.7	↑ 0.6				1.7	1.8	→ 0.1
5	Case management of anaemia and growth failure	0.8	1.1	↑ 0.3	1.0	1.7	↑ 0.7	0.1	1.5	↑ 1.4	1.3	2.2	↑ 1.0	1.3	1.5	↑ 0.3	1.4	1.6	↑ 0.23	1.3	1.9	↑ 0.6	1.8	2.0	↑ 0.3	1.4	1.6	→ 0.2				1.1	1.7	↑ 0.5
6	Case management of chronic conditions	2.0	1.7	↓ -0.3	1.0	1.7	↑ 0.7				1.6	2.0	↑ 0.4	1.5	1.6	→ 0.1	1.9			2.0	1.9	→ -0.1	2.0	2.1	→ 0.1	1.5	1.8	↑ 0.3				1.7	1.8	→ 0.2
7	Supportive care	1.9	2.0	→ 0.1	1.1	2.0	↑ 0.9	1.5	1.4	→ -0.1	2.1	2.3	→ 0.2	1.9	2.1	↑ 0.23	2.3	2.3	→ 0.0	1.8	2.4	↑ 0.7	1.5	2.2	↑ 0.7	1.8	2.2	↑ 0.4				1.8	2.1	↑ 0.3
8	Monitoring and follow up	1.0	2.3	↑ 1.3	1.0	2.0	↑ 1.0	1.2	1.8	↑ 0.6	2.0	2.4	↑ 0.4	1.5	1.8	↑ 0.3	1.8	1.7	→ -0.1	1.3	1.7	↑ 0.4	1.8	1.8	→ 0.0	1.5	1.6	→ 0.1				1.5	1.9	↑ 0.4
	**Maternal care**																																	
1	Care for normal labor and vaginal birth	1.1	1.5	↑ 0.4	1.2	1.6	↑ 0.4	1.1	1.5	↑ 0.4				2.4	2.0	↓ -0.4	2.6	2.2	↓ -0.4	2.2	2.1	→ -0.1	1.5	1.6	→ 0.1	1.5	1.6	→ 0.1	1.5	1.6	→ 0.1	1.8	1.9	→ 0.1
2	Care for caesarean section	1.9	1.9	→ 0.0	2.1	2.1	→ 0.0	2.0	1.5	↓ -0.5				1.9	2.0	→ 0.1	1.9	2.4	↑ 0.6	1.7	2.0	↑ 0.3	1.9	2.1	↑ 0.3	1.9	2.1	↑ 0.3	1.9	2.1	↑ 0.3	2.0	2.1	→ 0.2
3	Management of maternal complications	1.2	2.1	↑ 0.9	1.6	2.0	↑ 0.4	1.6	1.5	→ -0.1				1.7	2.1	↑ 0.4	1.5	2.3	↑ 0.7	1.5	1.7	→ 0.2	1.5	2.1	↑ 0.6	1.5	2.1	↑ 0.6	1.5	2.1	↑ 0.6	1.6	2.0	↑ 0.5
4	Monitoring and follow-up	1.7	1.8	→ 0.1	2.2	2.0	→ -0.2	2.0	1.2	↓ -0.8				2.5	2.8	↑ 0.3	2.3	2.5	↑ 0.3	2.2	1.8	↓ -0.4	2.0	2.5	↑ 0.5	2.0	2.5	↑ 0.5	2.0	2.5	↑ 0.5	2.1	2.1	→ 0.0
	**Newborn care**																																	
1	Newborn infant care	1.1	1.9	↑ 0.8	1.2	1.9	↑ 0.7	1.2	1.9	↑ 0.7				2.0	1.9	→ -0.1	1.5	2.2	↑ 0.7	1.3	2.1	↑ 0.7	2.0	1.7	↓ -0.3	1.7	2.2	↑ 0.5	1.7	2.2	↑ 0.5	1.6	2.0	↑ 0.4
2	Sick newborn care	1.2	1.8	↑ 0.7	1.2	2.0	↑ 0.9	1.2	2.0	↑ 0.9				1.5	1.8	↑ 0.3	1.6	1.9	↑ 0.3	0.3	1.5	↑ 1.2	1.4	1.1	↓ -0.3	1.3	2.4	↑ 1.1	1.3	2.4	↑ 1.1	1.2	1.8	↑ 0.7
3	Advanced newborn care	1.1	1.4	↑ 0.3	0.8	1.7	↑ 0.9	0.8	1.7	↑ 0.9				1.8	1.7	→ -0.1							1.5	1.6	→ 0.1	1.5	1.4	→ -0.1	1.5	1.4	→ -0.1	1.4	1.6	→ 0.2
4	Monitoring and follow-up	1.2	1.5	↑ 0.3	1.2	2.0	↑ 0.8	1.2	2.0	↑ 0.8				2.5	2.5	→ 0.0	2.0	1.8	↓ -0.3	1.3	1.8	↑ 0.5	1.5	2.0	↑ 0.5	1.2	2.3	↑ 1.1	1.2	2.3	↑ 1.1	1.7	2.0	↑ 0.3

[1] Colour coding: green- care meets international standards, little to no changes needed for quality improvement; yellow- care practices need some improvement to reach standards of care (care is suboptimal but without significant hazard to health); orange- need for substantial improvement to reach standards of care (suboptimal care with significant health hazards); red- need for substantial improvement (inadequate care and/ or harmful practices with severe health hazards). A score greater than 2.5 assigned with green colour, 1.8-2.4- yellow, 1-1.7- orange and less than 1- red. Categories of changes between baseline and endline: > 0.2 change in score was categorized as 'improvement'; ≤ -0.2 as 'deterioration' of services. The cut-offs followed the approach suggested in the WHO Quality Improvement manual to reflect the balance between possible random variation and documenting real improvement (or deterioration).

Panel C. Policies and organization of services

POLICIES AND ORGANIZATION OF SERVICES

HOSPITALS

		1			2			3			4			5		
		Baseline	Endline	Difference	Baseline	Endline	Difference	Baseline	Endline	Difference	Baseline	Endline	Difference	Baseline	Endline	Difference
	**Pediatric care**															
1	Infection prevention	2	1.7	-0.3	1.5	2.0	0.5	2.5	1.7	-0.8	1.9	2.7	0.8	1.8	2.6	0.9
2	Guidelines and audit	1	1.4	0.4	1.0	1.8	0.8	1.9	1.1	-0.8	1.8	2.2	0.4	1.9	2.1	0.23
3	Access to hospital care and continuity of care	2.0	2.0	0.0	2.0	2.0	0.0	2.0	1.1	-0.9	1.7	2.3	0.6	1.8	2.0	0.2
4	Respectful care	2.0	1.8	-0.2	2.0	2.3	0.3	1.5	1.4	-0.1	2.1	2.6	0.5	1.8	2.3	0.5
	**Maternal care**															
1	Infection prevention	1.5	2.0	0.5	1.1	2.1	1.0	1.0	1.6	0.6				2.8	1.8	-1.1
2	Guidelines and audit	1.0	1.2	0.2	1.5	1.8	0.3	1.0	1.7	0.7				2.0	1.8	-0.2
3	Access to hospital care and continuity of care	1.3	1.3	0.0	1.5	2.0	0.5	1.7	1.8	0.1				1.5	2.3	0.8
4	Respectful care	1.5	1.8	0.3	1.6	1.9	0.3	1.5	1.7	0.2				1.6	2.3	0.7
	**Newborn care**															
1	Infection prevention	0.8	2.0	1.2	1.1	2.1	1.0	0.8	1.4	0.6				2.8	1.8	-1.1
2	Guidelines and audit	1.0	1.7	0.7	1.5	1.8	0.3	1.0	1.8	0.8				2.0	1.8	-0.2
3	Access to hospital care and continuity of care	1.0	1.4	0.4	1.5	2.0	0.5	1.2	1.8	0.6				1.5	2.3	0.8
4	Mother and newborn rights	1.6	1.8	0.2	1.6	1.9	0.3	1.2	1.7	0.5				1.6	2.3	0.7

		6			7			8			9			10			Summary		
		Baseline	Endline	Difference	Baseline	Endline	Difference	Baseline	Endline	Difference	Baseline	Endline	Difference	Baseline	Endline	Difference	Baseline	Endline	Difference
	**Pediatric care**																		
1	Infection prevention	2.5	2.5	0.0	2.0	2.4	0.4	1.6	2.6	1.0	1.9	2.6	0.7				2.0	2.3	0.4
2	Guidelines and audit	2.0	2.1	0.1	1.3	1.9	0.6	1.6	2.3	0.7	1.0	1.3	0.3				1.5	1.8	0.3
3	Access to hospital care and continuity of care	2.1	2.2	0.1	2.0	2.3	0.3	2.0	2.5	0.5	2.0	2.2	0.2				2.0	2.1	0.1
4	Respectful care	2.3	2.5	0.2	2.2	2.6	0.4	1.5	2.1	0.6	1.5	2.0	0.5				1.9	2.2	0.3
	**Maternal care**																		
1	Infection prevention	2.6	1.8	-0.8	1.5	1.8	0.3	1.2	1.8	0.6	1.6	2.1	0.5	2.0	2.1	0.1	1.8	1.9	0.1
2	Guidelines and audit	2.3	1.8	-0.6	0.8	1.5	0.7	1.2	1.2	0.0	1.2	1.9	0.7	1.6	2.1	0.5	1.4	1.7	0.3
3	Access to hospital care and continuity of care	2.0	2.0	0.0	2.0	2.0	0.0	1.5	2.3	0.8	1.7	2.0	0.3	1.8	2.3	0.5	1.8	2.0	0.2
4	Respectful care	2.8	2.3	-0.5	1.6	1.3	-0.3	1.4	1.3	-0.1	1.2	1.3	0.1	1.0	2.1	1.1	1.6	1.8	0.2
	**Newborn care**																		
1	Infection prevention	2.6	1.8	-0.8	1.2	1.8	0.6	1.2	1.8	0.6	1.1	2.1	1.0	1.5	2.0	0.5	1.6	1.8	0.2
2	Guidelines and audit	2.3	1.8	-0.6	1.2	1.2	0.0	1.2	1.2	0.0	1.4	1.9	0.5	3.0	2.1	-0.9	1.5	1.7	0.2
3	Access to hospital care and continuity of care	2.0	2.0	0.0	1.5	2.3	0.8	1.5	2.3	0.8	1.6	2.0	0.4	2.0	2.3	0.3	1.5	2.0	0.5
4	Mother and newborn rights	2.8	2.3	-0.5	1.4	1.3	-0.1	1.4	1.3	-0.1	1.4	1.3	-0.1	2.0	2.0	0.0	1.6	1.7	0.2

Figure S2 in the **Online Supplementary Document**. Flow of the study: key assessments and quality improvement interventions

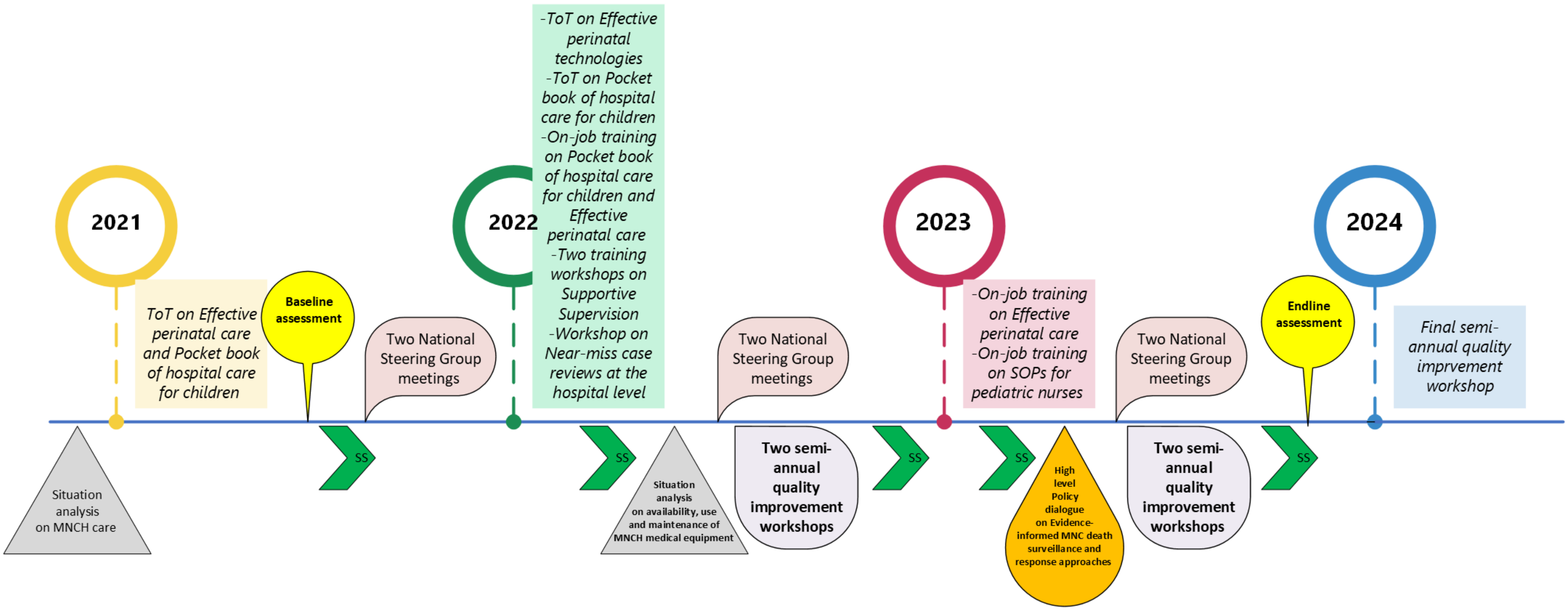

*MNC: maternal, newborn, child; SOP: standard operating procedures; ToT: training of trainers; SS: supportive supervision

Figure S3 in the **Online Supplementary Document**. Case management for respiratory infections

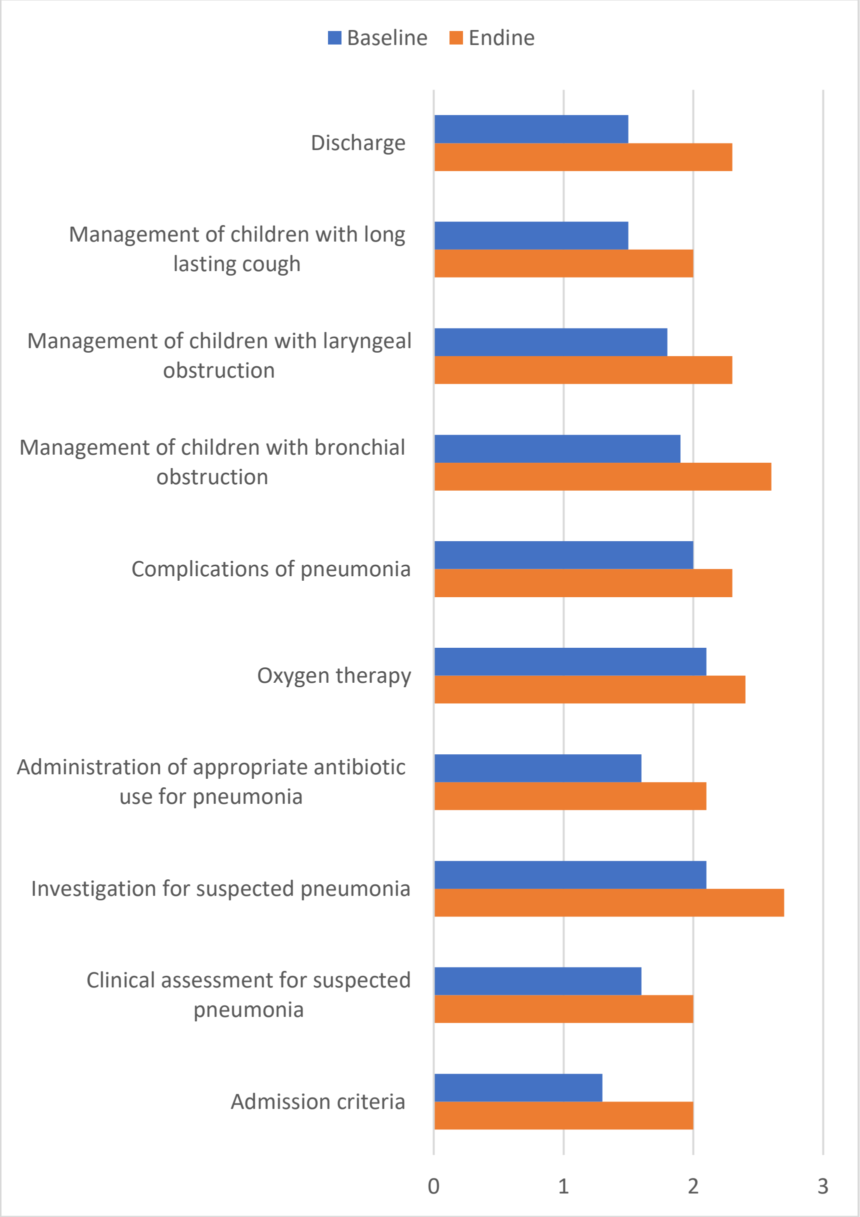

Figure S4 in the **Online Supplementary Document**. Case management for maternal care. Panel A. Management of maternal complications. Panel B. Caesarean section. Panel C. Care for normal labour and vaginal birth

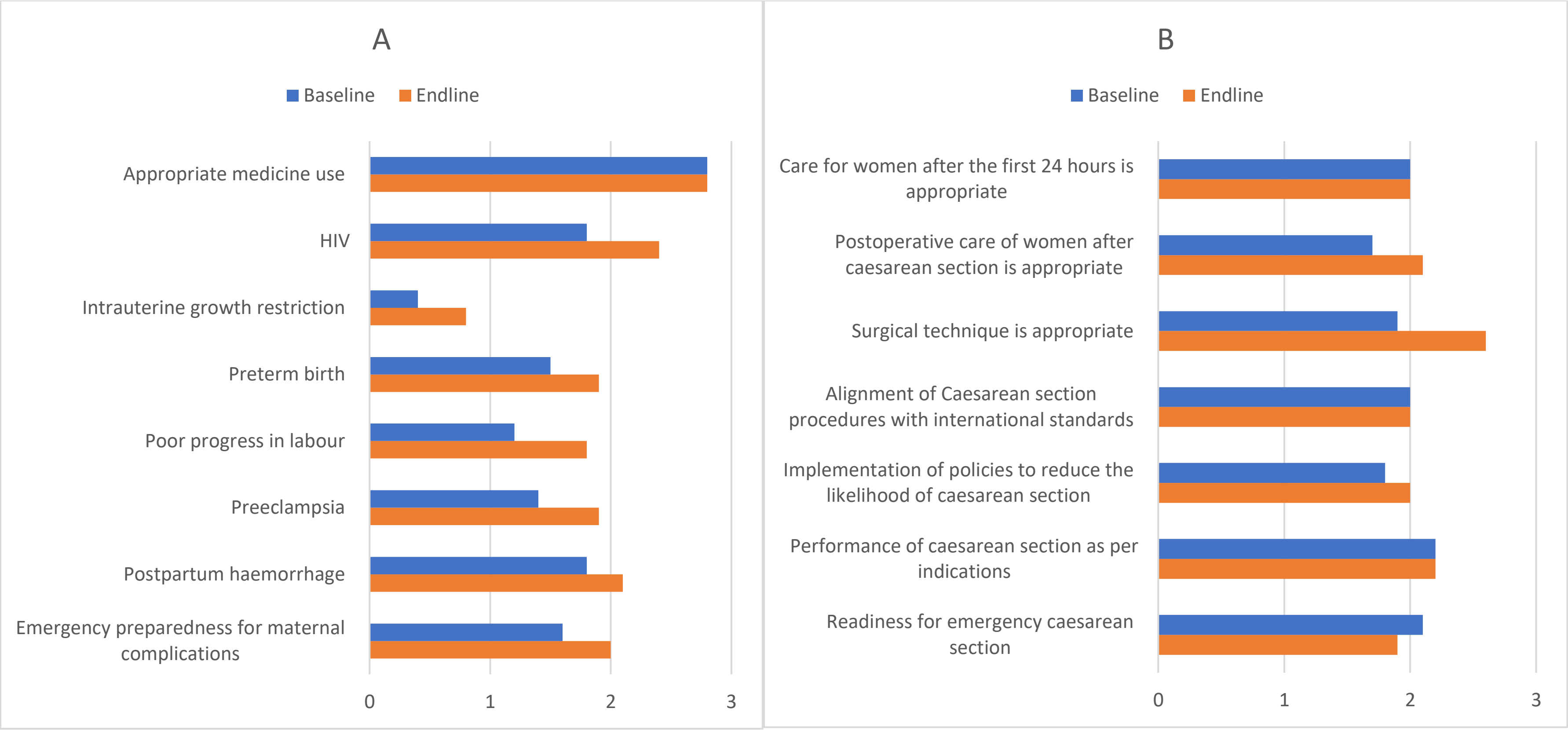

Figure S5 in the **Online Supplementary Document**. Case management for newborn care. Panel A. Routine newborn care. Panel B. Advanced newborn care

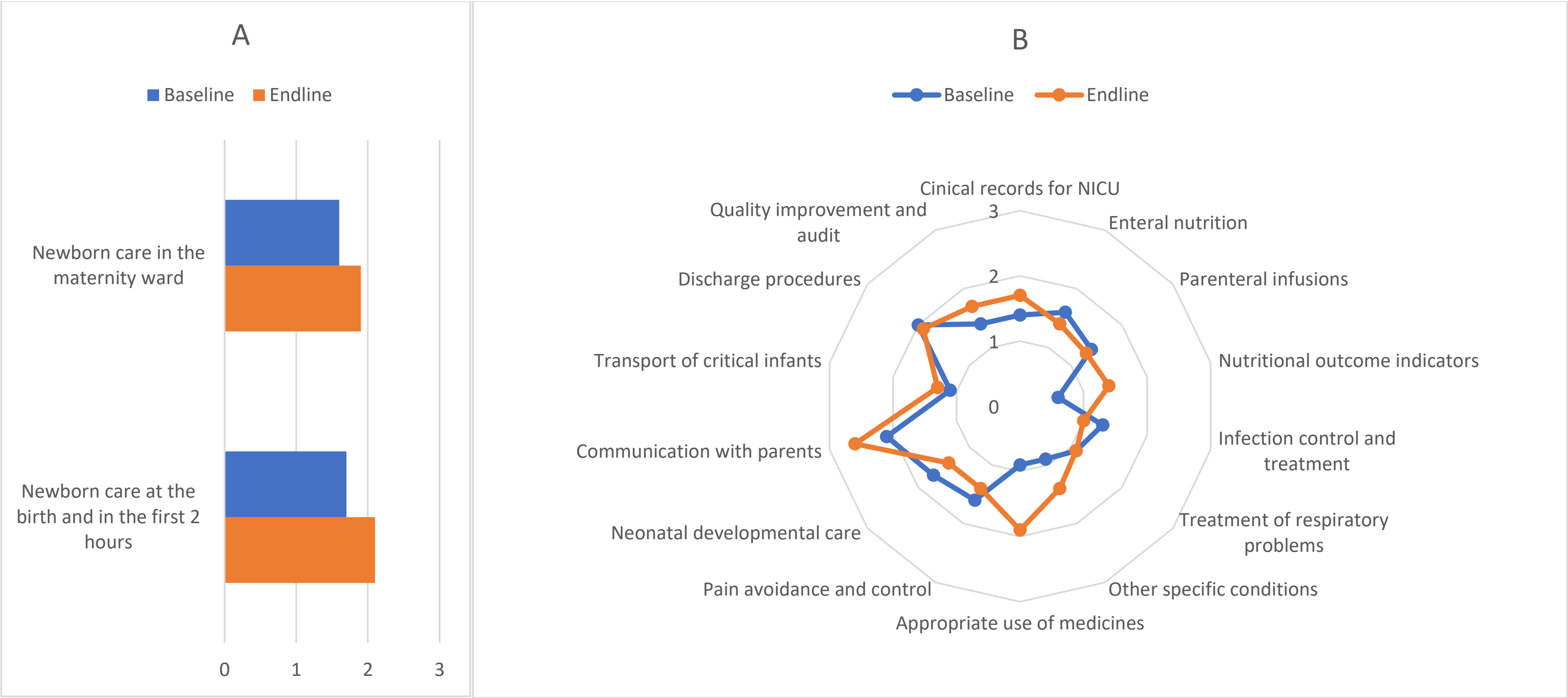
A
Baseline
Endline
Newborn care in the maternity ward
Newborn care at the birth and in the first 2 hours
0
1
2
3
B
Baseline
Endline
Cinical records for NICU
Enteral nutrition
Parenteral infusions
Nutritional outcome indicators
Infection control and treatment
Treatment of respiratory problems
Other specific conditions
Appropriate use of medicines
Pain avoidance and control
Neonatal developmental care
Communication with parents
Transport of critical infants
Discharge procedures
Quality improvement and audit
0
1
2
3

Figure S6 in the **Online Supplementary Document**. Hospital support services across areas. Panel A. Baseline. Panel B. Endline

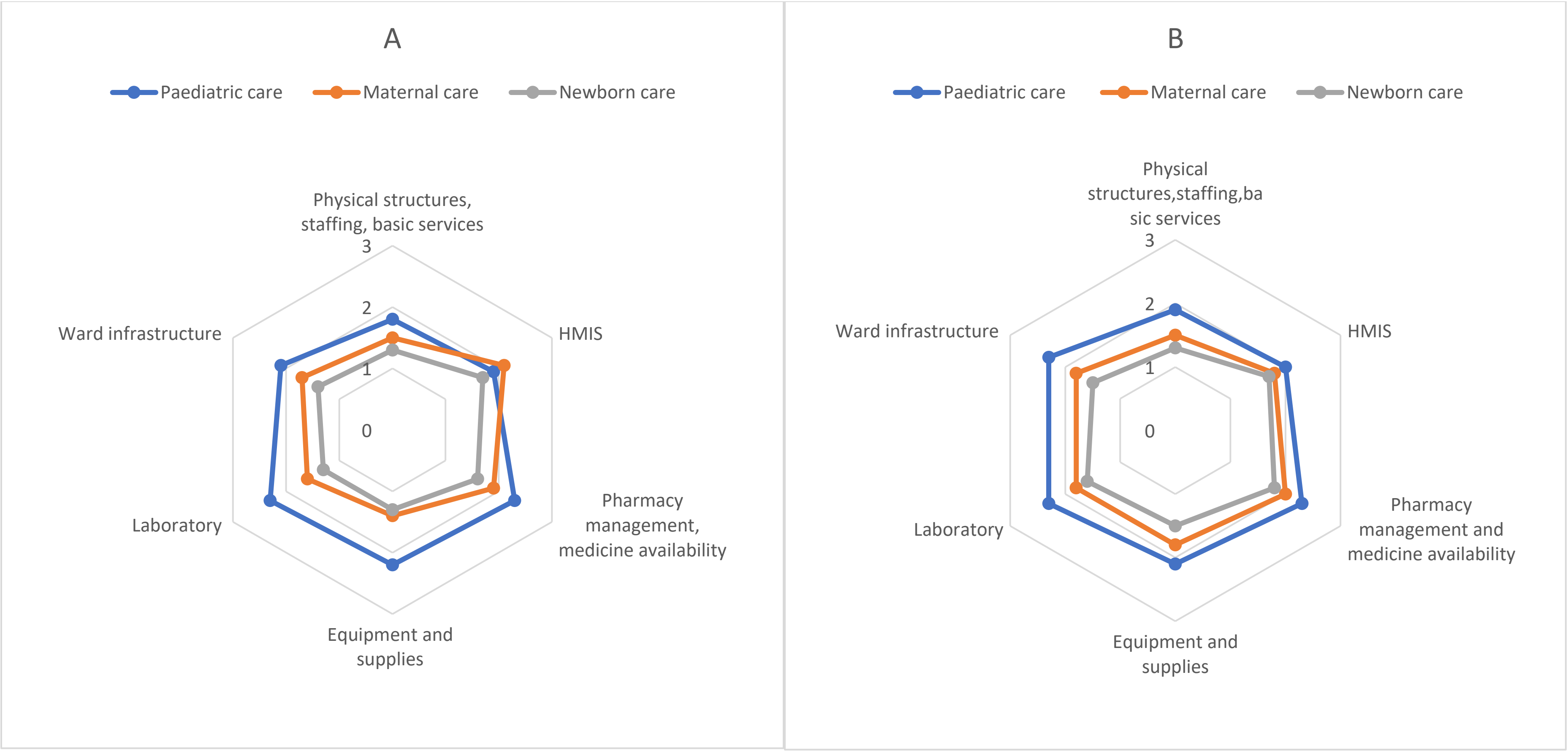

Supplement: Online Supplementary Document [file jogh-15-04256-s001.pdf]
